# Brain Connectivity and Prediction of Relapse after Cognitive-Behavioral Therapy in Obsessive–Compulsive Disorder

**DOI:** 10.3389/fpsyt.2015.00074

**Published:** 2015-05-20

**Authors:** Jamie D. Feusner, Teena Moody, Tsz Man Lai, Courtney Sheen, Sahib Khalsa, Jesse Brown, Jennifer Levitt, Jeffry Alger, Joseph O’Neill

**Affiliations:** ^1^Department of Psychiatry and Biobehavioral Sciences, University of California Los Angeles, Los Angeles, CA, USA; ^2^Laureate Institute for Brain Research, Tulsa, OK, USA; ^3^The University of Tulsa, Tulsa, OK, USA; ^4^Department of Neurology, University of California San Francisco, San Francisco, CA, USA; ^5^Department of Neurology, University of California Los Angeles, Los Angeles, CA, USA

**Keywords:** brain network, connectome, resting-state fMRI, graph theory, CBT

## Abstract

**Background:**

Intensive cognitive-behavioral therapy (CBT) can effectively reduce symptoms in obsessive–compulsive disorder (OCD). However, many relapse after treatment. Few studies have investigated biological markers predictive of follow-up clinical status. The objective was to determine if brain network connectivity patterns prior to intensive CBT predict worsening of clinical symptoms during follow-up.

**Methods:**

We acquired resting-state functional magnetic resonance imaging data from 17 adults with OCD prior to and following 4 weeks of intensive CBT. Functional connectivity data were analyzed to yield graph-theory metrics. We examined the relationship between pre-treatment connectome properties and OCD clinical symptoms before and after treatment and during a 12-month follow-up period.

**Results:**

Mean OCD symptom decrease was 40.4 ± 16.4% pre- to post-treatment (64.7% responded; 58.8% remitted), but 35.3% experienced clinically significant worsening during follow-up. From pre- to post-treatment, small-worldness and clustering coefficient significantly increased. Decreases in modularity correlated with decreases in OCD symptoms. Higher pre-treatment small-world connectivity was significantly associated with worsening of OCD symptoms during the follow-up period. Psychometric and neurocognitive measures pre- and post-treatment were not significant predictors.

**Conclusion:**

This is the first graph-theory connectivity study of the effects of CBT in OCD, and the first to test associations with follow-up clinical status. Results show functional network efficiency as a biomarker of CBT response and relapse in OCD. CBT increases network efficiency as it alleviates symptoms in most patients, but those entering therapy with already high network efficiency are at greater risk of relapse. Results have potential clinical implications for treatment selection.

## Introduction

Obsessive–compulsive disorder (OCD) is characterized by recurrent, intrusive, disturbing thoughts (obsessions), and/or stereotyped recurrent behaviors (compulsions) (1). Lifetime prevalence is 1–2%. Untreated OCD results in marked distress, and impaired functioning in social, occupational, and educational domains (2).

Cognitive-behavioral therapy (CBT) is an effective treatment for OCD (3). CBT typically consists of weekly outpatient treatment or intensive daily treatment over several weeks. Intensive CBT is particularly effective, resulting in sustained benefits (4, 5).

Notwithstanding, post-treatment relapse has been observed in roughly 20% of patients receiving CBT for OCD (6–12). Across studies, rates of relapse range widely from 0 to 50% (13). This variability may relate to differences in treatment parameters, patient characteristics, and even the definition of relapse (14). Although CBT is associated with significant symptom improvement and with lower relapse than a time-limited course of pharmacotherapy (13–19), many clinical questions remain. One important question is: what factors help predict who will remain in remission and who will relapse after treatment?

While there is some evidence for predictors of short-term response to treatment (20–22), fewer studies have addressed the equally important question of sustained response, or, conversely, worsening after treatment (23, 24). Several naturalistic follow-up studies have attempted to identify predictors of symptom course. One study followed a cohort for 21 months after a 12-week randomized trial of group CBT or fluoxetine (25). Comorbid affective disorders, any comorbid psychiatric condition, and illness duration were associated with higher post-treatment OCD symptoms. A study that followed an OCD cohort treated with 12 sessions of group CBT for 2 years found that full remission of symptoms at the end of treatment protected against relapse (26). Another study followed patients for 6 years after receiving CBT with clomipramine or placebo. Greater amount of CBT, better homework compliance, and improvement at the end of treatment were associated with better outcomes (9). Likewise, in a meta-analysis of pediatric OCD, severity of illness, comorbid diagnoses, and poor initial treatment response were associated with worse longitudinal course (27). However, another study that followed patients 6–8 years after CBT plus fluvoxamine or placebo did not find an association between short-term treatment response and long-term outcome (28).

Prognostic factors that could serve as biomarkers of relapse after treatment would have potential use in clinical decision-making. For example, factors such as psychometric scores or neurocognitive profiles; or measurements of brain activity, connectivity, morphometry, or neurochemical profiles associated with relapse could be identified prior to a particular treatment and could be used to determine whether an alternative treatment should be pursued.

Measurement of brain activity is a logical target for identification of putative biomarkers of clinical outcome, since multiple studies associate OCD with hyperactivity in frontostriatal systems (29, 30). Resting-state functional magnetic resonance imaging (rsfMRI) is increasingly used to understand functional brain connectivity in psychiatric disorders. Analyses of rsfMRI data allow for examination of spontaneous fluctuations in the blood–oxygen level-dependent (BOLD) signal, revealing underlying intrinsic connectivity networks. rsfMRI studies in OCD have detected aberrant connectivity in frontostriatal and parietal regions that may contribute to the emergence and severity of OCD symptoms (31, 32).

Graph theory is an analysis technique used to study rsfMRI that provides information about the topology of intrinsic brain connectivity networks (33). Graph theory provides quantitative analyses of complex brain networks as a whole, rather than being limited to discrete pairs of regions. To date, there have been only two graph-theory studies of OCD. One found abnormally low connectivity in posterior temporal cortex, and abnormally high connectivity in middle cingulate, precuneus, thalamus, and cerebellum (34). A second study found abnormally low clustering coefficient, small-worldness, and local efficiency in OCD at baseline (35). Further, medication treatment was associated with increased small-worldness, clustering coefficient, local efficiency, and modularity.

The goal of the current study was to investigate, for the first time, if pre-treatment functional network connectivity measures predict worsening of OCD symptoms subsequent to CBT treatment. An additional goal was to determine the effects of intensive CBT on global brain network connectivity using graph theory. We emphasized pre-treatment rather than post-treatment values in order to uncover potential biomarkers that could provide useful input to early clinical decision-making about selection of treatment modality. We determined graph-theory metrics of rsfMRI network connectivity in individuals with OCD prior to and following 4 weeks of intensive CBT. We then followed individuals for up to 12 months post treatment, and determined the relationship between pre-treatment connectivity metrics and follow-up clinical symptomatology.

We hypothesized that intensive CBT would result in increases in functional network efficiency – mean clustering coefficient, local efficiency, and small-worldness – and modularity, based on the previous study of OCD treatment (35). In addition, we predicted a significant relationship between these graph-theory metrics pre-treatment and worsening of core OCD symptoms at follow-up. Moreover, we hypothesized that these connectivity measures, being less subjective and effort-dependent, would demonstrate stronger associations with follow-up clinical status than psychometric and neurocognitive variables.

## Materials and Methods

### Participants

Seventeen right-handed adults ages 21–50 diagnosed with DSM-IV OCD (36) participated. Participants were recruited from UCLA clinics, local psychiatrists and psychotherapists, flyers, and Internet advertisements. All provided written informed consent, and the UCLA Institutional Review Board approved the study. Diagnoses of OCD participants were established through detailed interviews conducted by one of the authors (JDF), who has clinical experience with this population. Primary OCD and comorbid diagnoses were determined using the ADIS-IV-Mini (37). Participants were eligible if they scored ≥16 on the Yale–Brown Obsessive–Compulsive Scale (YBOCS) (38). Exclusion criteria included a psychotic disorder, bipolar disorder, lifetime substance dependence, or attention-deficit hyperactivity disorder. Comorbid anxiety disorders were allowed, as long as OCD was the primary diagnosis. Comorbid major depressive disorder (MDD) or dysthymic disorder was allowed, but individuals were excluded if the ADIS-IV clinical significance rating was 6 or higher (severe). Unmedicated or medicated participants were included, but serotonin reuptake inhibitors were the only class of medication allowed. In addition, they could not have had any changes (dose or agent) to their medication within 12 weeks prior to enrollment. Other exclusion criteria included IQ <80 on the Wechsler Abbreviated Scales of Intelligence (WASI) (39) and medical conditions that affect cerebral metabolism (e.g., thyroid disorders, diabetes). We also excluded those with prior courses of CBT for OCD consisting of ≥30 sessions, in order to minimize the possibility that such patients may already have experienced putative brain changes induced by CBT. Demographics and psychometrics are reported in Tables 1 and 2, and Table S1 in Supplementary Material. Three individuals were taking stable doses of serotonin reuptake inhibitors (two fluoxetine, one escitalopram) and 14 were unmedicated.

**Table 1:** **Demographics of OCD participants**.

Demographics	
Total participants (*N*)	17
Gender (F/M)	9/8
Age (years)	34.0 ± 9.43
Years of education	16.06 ± 2.19
IQ (WASI)	109.41 ± 6.14
No comorbidities/comorbidities	2/15
Medicated/unmedicated during intensive CBT phase	3/14
Medication treatment only during follow-up phase	*N* = 4
CBT only during follow-up phase	*N* = 8
Medications and CBT during follow-up phase	*N* = 2
No treatment during follow-up phase	*N* = 3
Mean follow-up duration (mo.)	7 ± 4.53

*WASI, Wechsler Abbreviated Scales of Intelligence; CBT, cognitive-behavioral therapy*.

**Table 2:** **Psychometrics of OCD participants**.

Psychometric	Pre-treatment	Post-treatment	Follow-up	Statistic	df	*P* value
YBOCS	23.12 ± 3.04^a^	13.76 ± 4.25^b^	15.47 ± 6.65^c^	*F* = 26.62	1.44, 22.98	<0.001
HAMA	11.94 ± 4.78^e^	8.06 ± 4.66^f^	9.12 ± 4.92^g^	*F* = 5.85	2, 32	0.007
MADRS	12.12 ± 5.51	9.53 ± 9.71	10.00 ± 4.86	*F* = 0.85	1.43, 22.89	0.40
GAS	59.06 ± 6.65^h^	74.06 ± 13.66^i^	73.65 ± 10.95^j^	*F* = 14.58	2, 32	<0.001
Stroop interference	54.59 ± 7.37	54.47 ± 7.88	N/A	*t* = 0.10	16	0.92
Sheehan (%)	N/A	N/A	27.25 ± 20.56	N/A	N/A	N/A
QLESQ (%)	N/A	N/A	59.56 ± 10.13	N/A	N/A	N/A

*YBOCS, Yale Brown Obsessive–Compulsive Scale; HAMA, Hamilton Anxiety Scale; MADRS, Montgomery Åsberg Depression Rating Scale; GAS, Global Assessment Scale; Sheehan, Sheehan Disability Scale; QLESQ, Quality of Life Enjoyment and Satisfaction Questionnaire Short Form*.

*^a^ vs. ^b^: *P* < 0.001; ^a^ vs. ^c^: *P* = 0.001; ^b^ vs. ^c^: *P* = 0.673; ^e^ vs. ^f^: *P* = 0.011; ^e^ vs. ^g^: *P* = 0.079; ^f^ vs. ^g^: *P* = 1; ^h^ vs. ^i^: *P* < 0.001; ^h^ vs. ^j^: *P* < 0.001; ^i^ vs. ^j^: *P* = 1*.

### Psychometric evaluations

An independent evaluator, not involved in treatment or initial assessments, administered the psychometric instruments post-treatment and during the follow-up period. After 4 weeks of intensive CBT, participants returned to care as usual, which may have included medication treatment and/or psychotherapy (CBT or other), or no treatment. Thus, the follow-up period was naturalistic rather than controlled.

The primary outcome was the YBOCS (38). The YBOCS is a reliable and valid semi-structured clinical interview assessing OCD severity and change over time. It has excellent psychometric properties and sensitivity to treatment. Treatment response was defined as a ≥35% reduction of YBOCS total score and a Clinical Global Improvement (CGI) score of ≤2; remission was defined as a YBOCS score of ≤14 (40). Secondary outcome measures pre- to post-treatment included the Hamilton Anxiety Scale (HAMA) (41) and the Montgomery-Åsberg Depression Rating Scale (MADRS) (42). A general rating of functionality and of social and occupational performance was provided by the Global Assessment Scale (GAS) (43).

We also administered the Stroop Color–Word Interference Task (44) to measure response inhibition. Within this neurocognitive task, the interference trial requires the participant to inhibit a pre-potent response in order to provide a correct answer. We used interference scores as a secondary independent variable of interest.

### Clinical procedures

All participants underwent intensive CBT with an individual therapist, consisting of 90-min sessions, 5 days per week, for 4 weeks. (For additional details see Supplementary Material.)

Following treatment, participants underwent the Stroop task and psychometric evaluations. They were then followed over a period of 1–12 months (mean 7 ± 4.53). Follow-up assessments included the YBOCS, MADRS, HAMA, the Sheehan Disability Scale (45), and the Quality of Life Enjoyment and Satisfaction Questionnaire Short Form (Q-LES-Q) (46).

### fMRI acquisition

Participants were scanned prior to and following 4 weeks of CBT. Magnetic resonance data were acquired at 3 T using a Siemens Trio with 12-channel headcoil. Whole-brain fMRI was collected using a 7-min echo-planar imaging (EPI) sequence (TR/TE = 2000/25 ms, flip angle = 78°, voxels 3 mm^3^, 35 interleaved slices with 1-mm gap). Participants were instructed to rest with eyes closed, to remain as still as possible, and not to sleep. High-resolution T1-weighted whole-brain structural MRI was acquired using an axial MPRAGE sequence (TR/TE = 1900/3.26 ms, voxels 1 mm^3^), used for registration of BOLD data.

### rsfMRI processing

Functional data were preprocessed using FMRI Software Library (FSL) version 5.0.4^1^. To allow for magnetization equilibrium, we discarded the first two images. Data were slice-time corrected, were motion-corrected (MCFLIRT), and band-pass filtered (0.009–0.08 Hz). A seven degrees-of-freedom (DOF) transform was used to register each participant’s functional image to the MPRAGE, and a 12-DOF transform was used to register the MPRAGE to Montreal Neurological Institute (MNI) standard atlas space. All images were inspected for proper registration to the MNI template. Data were then resampled to 2-mm space. White matter, CSF, and temporal derivatives were removed by linear regression, as were six head motion parameters. Motion was assessed using DVARS (root mean squared change in BOLD signal from volume to volume), and one participant was excluded whose DVARS exceeded 25% (47). There was no difference in motion pre-and post-treatment, as indicated by DVARS measured for each subject across the time-series (9.69 ± 1.51 pre-CBT and 9.29 ± 1.16 post-CBT; *P* = 0.33) (Figure S2 in Supplementary Material).

### Brain network extraction

**Figure 1:**
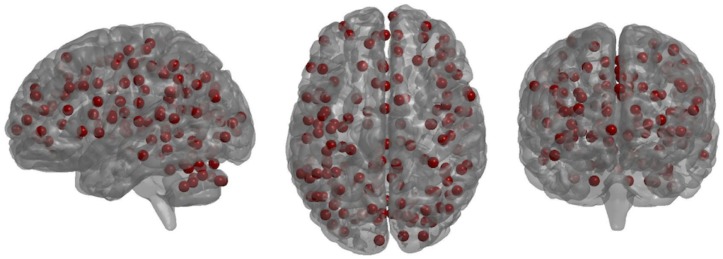
**Sagittal, axial, and coronal views of the 160 nodes used for the graph-theory analysis**.

One hundred sixty functionally defined nodes were used, covering the whole brain, as previously described (48) (Figure 1). Nodes were 10 mm in diameter and non-overlapping. Applying these nodes to each individual’s rsfMRI time-series data, all pairwise partial correlation coefficients between nodes were calculated. We chose partial over full correlations as the former make topological parameters less sensitive to motion, especially at higher density thresholds (49). (We additionally performed Pearson full correlation analyses – see Supplementary Material.) Using the Brain Connectivity toolbox http://www.brain-connectivity-toolbox.net/, we derived weighted, undirected graphs, or functional connectivity matrices, for each participant.

In graph theory, a network comprises “nodes” (here, functionally defined ROIs from five meta-analyses of fMRI activation studies) and the connections or “edges” between them (in this case, partial correlation values). The *clustering coefficient* of a node is the ratio of the number of actual connections among its first-degree neighbors to the number of all possible such connections. Thus, a high clustering coefficient for a node indicates that its neighbors are strongly inter-connected. *Mean clustering coefficient* is the average of the clustering coefficient for all nodes in the network; high values may confer greater local efficiency of information transfer in a network (33). *Global efficiency* is mathematically defined by averaging the inverse shortest path lengths across all node pairs. (Path length is the minimum number of intermediate nodes needed to pass through in order to link any node pair.) High global efficiency represents high overall capacity for parallel information transfer and integrated processing (50). *Small-worldness* is mean clustering coefficient divided by characteristic path length. *Modularity* measures how strongly nodes in a community interconnect in comparison to nodes in a random graph. Thus, the higher the modularity of a given community structure, the less likely it is the result of chance alone. Modularity quantifies the degree to which the network may be subdivided into such clearly delineated modules. In the brain, balance between segregation of specialized systems (modules) and integration across systems is essential for efficient information processing and rapid information transfer within and between networks (51, 52).

From the connectivity matrices, we calculated small-world parameters (including mean clustering coefficient, small-worldness, local and global efficiencies), and modularity, to examine global network topologies. For small-world analysis, we used the sparsity threshold *S* to define the small-world regime (0.10 ≤ *S* ≤ 0.50). Each small-world attribute was compared with those of 100 random networks to produce a normalized value, and areas under the curve (AUC) were calculated for statistical comparisons.

### Statistical analyses

We tested relationships between pre-treatment graph-theory network measures and change in OCD symptoms during follow-up using multiple linear regression in SPSS^®^. All data were checked for normality of distributions and for outliers. We tested small-worldness, mean clustering coefficient, global efficiency, local efficiency, and modularity. The primary outcome was change in YBOCS from post-treatment to the follow-up time point. For the prediction of follow-up score analyses, we controlled for the number of months post-treatment at which follow-up data were acquired, as well as for number of months of medication treatment and number of CBT sessions (if any) in the follow-up period, and for motion (DVARS for the pre-CBT scan). Because small-worldness is a function of clustering coefficient and (the inverse of) global efficiency, and local efficiency and clustering coefficient are related and therefore non-independent, we use a Bonferroni-corrected α threshold of 0.05/3 = 0.017 for the independent statistical metrics (clustering coefficient, global efficiency, and modularity).

Secondary analyses examined whether clinical (HAMA and MADRS scores) or neurocognitive (Stroop interference scores) variables pre- or post-treatment significantly predicted follow-up YBOCS score changes. A Bonferroni-corrected α threshold of 0.05/6 = 0.0083 was used for statistical significance.

To assess differences pre- to post-treatment within-group, we compared AUC values obtained across the range of sparsities. We applied multiple linear regression before computation to remove the confounding effects of motion (DVARS) for each graph-theory metric. We performed paired *t*-tests, or one-sample permutation tests in R^2^, for data that were normally or non-normally distributed, respectively. A significance threshold of α = 0.017, two-tailed, was applied, as above.

As exploratory analyses, we tested for pre- to post-CBT changes at the level of individual regions for all 160 nodes, for the graph-theory metrics node degree, clustering coefficient, local efficiency, betweenness centrality, and edge betweenness centrality. We used false discovery rate (FDR) to correct for multiple comparisons for each metric, with a *q* value set at.05/4 = 0.0125 (to account for four independent tests of metrics, as clustering coefficient and local efficiency are related) as the significance threshold.

## Results

### Treatment response and follow-up clinical data

Follow-up data were available for all 17 participants. Mean duration of follow-up was 7 ± 4.53 months. Four participants received medication only in the follow-up period, eight were treated with CBT only, two with medications and CBT, and three received no treatment (Table 1).

All 17 participants completed treatment. Mean YBOCS scores decreased 40.4 ± 16.4% from pre- to post-treatment [*t*(16) = 10.00, *P* < 0.0001] (Table 2). Eleven (64.7%) were responders and 10 (58.8%) achieved remission.

There was a mean increase in YBOCS from post-treatment to follow-up of 13.98 ± 50.91%. Six (35.3%) experienced clinically significant worsening of symptoms during follow-up consisting of ≥5 points worsening on YBOCS (53).

### Pre- to post-treatment changes in graph-theory global network metrics

**Figure 2:**
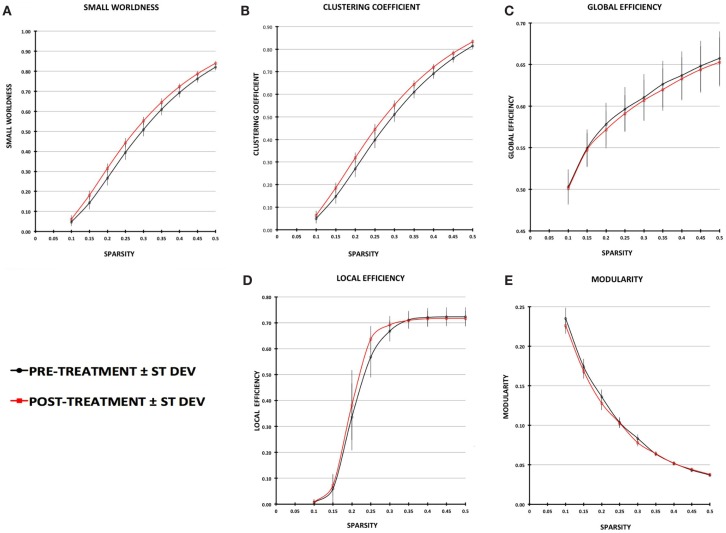
**Plot of graph-theory metrics pre- and post-treatment, derived from partial correlation matrices across sparsity levels from 0.1 to 0.5: (A) small-worldness, (B) clustering coefficient, (C) global efficiency, (D) local efficiency, (E) modularity**. Error bars are ±1 SD.

**Figure 3:**
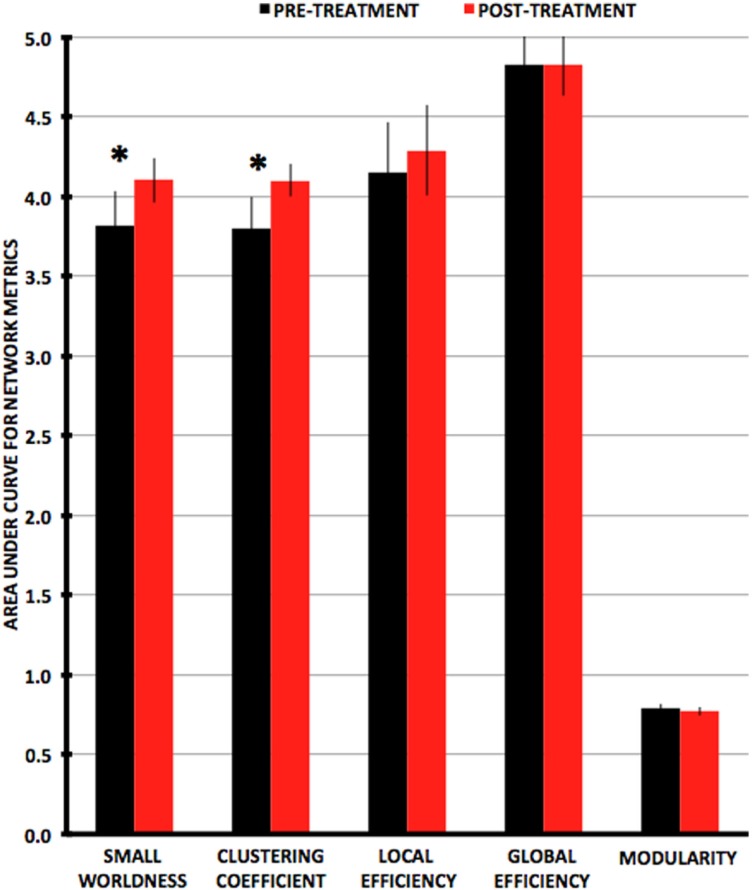
**Plots of average values for area-under-the-curve network metrics with pre-treatment in black and post-treatment in red**. Metrics that are significantly different (*P* < 0.0167) pre- vs. post-treatment are indicated by an asterisk.

Small-worldness AUC significantly increased from pre- to post-treatment (*P* = 0.012), as did mean clustering coefficient (*P* = 0.0093) (Figures 2 and 3). There were non-significant increases in local efficiency (*P* = 0.070), non-significant decreases in modularity (*P* = 0.12), and non-significant decreases in global efficiency (*P* = 0.18). *Post hoc* analyses excluding medicated individuals resulted in similar significant findings, with the exception that modularity significantly decreased from pre- to post-treatment (*P* = 0.012) (see Supplementary Material).

**Figure 4:**
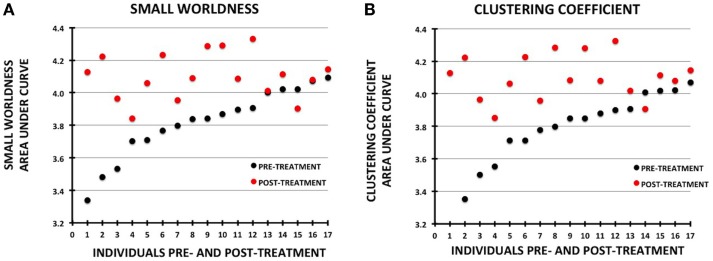
**Plots showing change in metrics for individual participants pre- vs. post-treatment**. The data for each plot were sorted by the pre-treatment values. Pre-treatment values are shown with black circles and post-treatment as red circles: **(A)** small-worldness, **(B)** clustering coefficient.

Changes in small-worldness were relatively consistent across the sample, with 16 of 17 participants demonstrating increases pre- to post-treatment (although 2 were minimally changed) (Figure 4). Mean clustering coefficient increased in 15 of 17.

As *post hoc* analyses, we tested partial correlations between changes in network metrics and changes in OCD symptom scores, controlling for motion (mean pre- and post-CBT DVARS). We used a Bonferroni-corrected α threshold of 0.05/3 = 0.0167 for statistical significance. Change in modularity was significantly correlated with change in YBOCS from pre- to post-treatment (*r* = 0.64; *P* = 0.007). That is, decreases in modularity were associated with decreases in OCD symptoms. Correlations were non-significant for changes in small-worldness (*r* = −0.42; *P* = 0.10), mean clustering coefficient (*r* = −0.42; *P* = 0.10), global efficiency (*r* = −0.13; *P* = 0.63), and local efficiency (*r* = −0.13; *P* = 0.64).

### Exploratory node-level analyses of pre- to post-treatment changes in graph-theory metrics

There were no significant differences for any nodes pre- to post-CBT for node degree, clustering coefficient, local efficiency, betweenness centrality, and edge betweenness centrality, after FDR correction (see Table S2 in Supplementary Material).

### Graph-theory metric predictions of follow-up OCD symptom scores

**Figure 5:**
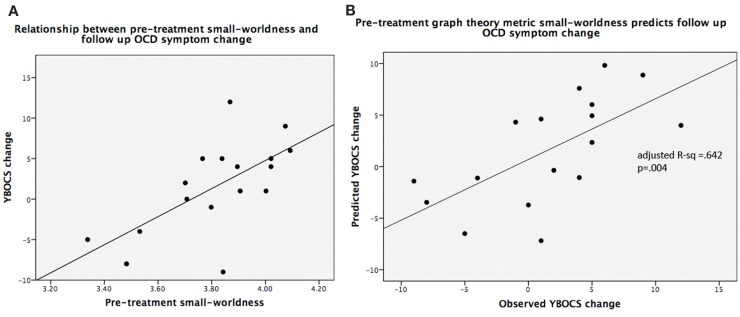
**(A)** Scatterplot of small-worldness values vs. observed YBOCS changes in the post-treatment follow-up period; **(B)** linear regression results of predicted YBOCS changes vs. observed YBOCS changes. Note: **(A)** is shown purely to display the relationship between the two variables, while **(B)** depicts the linear regression results that control for the covariates of non-interest.

Pre-treatment small-worldness was significantly associated with follow-up change in YBOCS scores (adjusted *R*^2^ = 0.642; *F*_5,11_ = 6.75, *P* = 0.004) (Figure 5), as was pre-treatment mean clustering coefficient (adjusted *R*^2^ = 0.647; *F*_5,11_ = 6.87, *P* = 0.004). For both metrics, higher pre-treatment values were associated with worsening on YBOCS. Global efficiency (adjusted *R*^2^ = 0.21; *F*_5,11_ = 1.83, *P* = 0.19), local efficiency (adjusted *R*^2^ = 0.074; *F*_5,11_ = 1.25, *P* = 0.35), and modularity (adjusted *R*^2^ = −0.032; *F*_5,11_ = 0.90, *P* = 0.51) were not significant predictors of follow-up score changes.

Reanalysis of the data after excluding the three participants taking medications during the pre- to post-treatment period revealed that pre-treatment small-worldness continued to be significantly associated with follow-up change in YBOCS (adjusted *R*^2^ = 0.661; *F*_5,8_ = 6.08, *P* = 0.013), as was pre-treatment mean clustering coefficient (adjusted *R*^2^ = 0.664; *F*_5,8_ = 6.15, *P* = 0.013). In addition, global efficiency was associated with follow-up change in YBOCS (adjusted *R*^2^ = 0.593; *F*_5,8_ = 4.79, *P* = 0.025), although not surviving correction for multiple comparisons; lower pre-treatment global efficiency was associated with worsening on YBOCS.

As *post hoc* exploratory analyses, we investigated the relationships between the graph-theory metrics and additional clinical measures of anxiety (HAMA), depression (MADRS), quality of life (Q-LES-Q), and general functioning (Sheehan) at follow-up. We used a Bonferroni-corrected α threshold of 0.05/12 = 0.004 (four clinical variables × three independent graph-theory metrics). None of the global graph-theory metrics were significantly associated with HAMA, MADRS, Q-LES-Q, or Sheehan scores.

### Clinical and neurocognitive predictors of follow-up OCD symptom scores

Yale–Brown Obsessive–Compulsive Scale scores pre-treatment (adjusted *R*^2^ = −0.002, *F*_4,12_ = 0.99, *P* = 0.49) or post-treatment (adjusted *R*^2^ = 0.009, *F*_4,12_ = 1.04, *P* = 0.43) did not predict follow-up YBOCS. HAMA scores pre-treatment (adjusted *R*^2^ = 0.15, *F*_4,12_ = 1.73, *P* = 0.21) and post-treatment (adjusted *R*^2^ = 0.009, *F*_4,12_ = 1.04, *P* = 0.43) did not predict follow-up YBOCS scores, nor did MADRS scores pre-treatment (adjusted *R*^2^ = 0.002, *F*_4,12_ = 1.01, *P* = 0.44) or post-treatment (adjusted *R*^2^ = 0.29, *F*_4,12_ = 2.53, *P* = 0.10). Stroop interference scores pre-treatment (adjusted *R*^2^ = −0.001, *F*_4,12_ = 0.99, *P* = 0.45) and post-treatment (adjusted *R*^2^ = 0.002, *F*_4,12_ = 1.01, *P* = 0.44) did not predict follow-up YBOCS. Number of months on medications and number of follow-up CBT sessions either modeled separately (adjusted *R*^2^ = −0.10, *F*_2,14_ = 0.26, *P* = 0.77 for medications; adjusted *R*^2^ = 0.070, *F*_2,14_ = 1.60, *P* = 0.24 for CBT) or together (adjusted *R*^2^ = 0.075, *F*_3,13_ = 1.43, *P* = 0.28) did not significantly predict YBOCS scores.

## Discussion

This is the first study to identify neuroimaging predictors of longitudinal clinical course subsequent to treatment for OCD. It is also the first to test effects of CBT treatment on brain network connectivity using graph theory. Results suggest that small-world network efficiency increases in almost all patients during CBT, but that patients with higher pre-treatment efficiency are more prone to relapse. Thus, increasing network efficiency may be CBT-associated pathways to recovery from OCD, but patients with already high network efficiency may be at greater risk of relapse of OCD symptoms.

### Prediction of longitudinal clinical status

As hypothesized, mean clustering coefficient and small-worldness significantly predicted follow-up OCD symptom severity, although, contrary to prediction, global efficiency and modularity did not. Mean clustering coefficient and small-worldness were better predictors than clinical measures including OCD symptom severity and anxiety and depression before or after treatment. Likewise, they performed better than a neurocognitive measure of response inhibition (Stroop), which was not significantly associated with follow-up scores. Surprisingly, months of medication and number of CBT therapy sessions in the follow-up period also did not predict OCD symptom severity. Thus, global measures of brain connectivity show the strongest relationship with symptom course subsequent to intensive CBT over any clinical or neurocognitive measure tested.

### Changes in network connectivity with treatment

As hypothesized, results demonstrated significant pre- to post-treatment increases in individuals with OCD in small-worldness and mean clustering coefficient, although increases in local efficiency were not significant. Modularity, contrary to prediction, did not significantly increase from pre- to post-treatment. These observations of increases in small-worldness and mean clustering coefficient, but no increases in global efficiency, replicate findings in a study of OCD treatment with medication (35), although we did not observe similar changes in modularity. Therefore, there may be non-specific effects of treatment on small-world efficiency measures, but divergent effects of CBT and medications on community network structure.

Interestingly, those with lower small-worldness and lower mean clustering coefficient pre-treatment were less likely to experience worsening of their OCD symptoms in the follow-up period. These values increased significantly with treatment, although the greatest increases were for those with low pre-treatment values. This suggests that while CBT may improve network efficiency, this effect is strongest in those who start with low brain network efficiency. Moreover, these individuals do better longitudinally, in terms of sustaining or even further decreasing OCD symptom severity.

Small-world organization of brain connectivity provides for efficiency in local information processing; high local neighborhood clustering and long-distance connections offer a high degree of efficient global communication across the network and integration from different sub-specialized neighborhoods (33, 54–56). Since small-worldness is a function of high clustering coefficient and low characteristic path length (inverse of global efficiency), in the current study, the increases in small-world efficiency subsequent to treatment are driven by increases in mean clustering coefficient, while global efficiency remains relatively unaffected. Clustering coefficient is a measure of functional segregation; higher mean clustering coefficient means that, on average, each region’s neighbors are more highly connected with each other. This can also be thought of as more densely inter-connected local networks of brain regions that confer segregated neural processing (57), for example, perhaps those involved in specialized brain functions such as attunement to specific auditory frequencies, or consolidation of fear learning in local limbic circuits. Thus, in our study, the finding of increased small-worldness as a result of increased clustering coefficient means that the brain networks have increased their overall efficiency through enhanced regional processing efficiency conferred by more dense connections, while retaining the same long distance connections (no significant changes in global efficiency) that are important for integration of information (57). Since increases in small-worldness and mean clustering coefficient were not correlated with improvements in OCD symptoms pre- to post-treatment, the mechanism of CBT in effecting improvements in network efficiency could be independent from direct effects on obsessive thoughts and compulsive behaviors.

One possible mechanism could relate to general effects that CBT may have in helping patients organize their thoughts, feelings, and behaviors, and improve their ability to be a “rational observer” of these phenomena in their mind. This may relate to improvements through extended psychoeducation and practice in monitoring their experience, which can deepen their understanding of the mental processes inherent to OCD. Improvement in these areas, which provides the foundation and enhances insight to further address obsessions and compulsions with the behavioral (exposure and response prevention) stage of treatment, might confer increases in brain network efficiency. This aspect of CBT might allow for disparate, functionally segregated processing of emotions, thought patterns, sensory–motor experiences, and planned/executed behaviors to become more integrated into conscious awareness, which subsequently may be reflected in changes in small-world properties. Those with already high network efficiency when treatment begins may represent a more refractory group in the long-term, as they already may be employing these techniques but have not improved or remained well; this is the group that is at most risk for relapse. This conjectural interpretation, however, needs to be further explored and confirmed in future studies that could, for example, test effects of these components of psychoeducation and early stages of CBT, in isolation from its behavioral components.

Decreases in modularity with treatment were correlated with improvements in OCD symptoms. Optimal modularity implies an ideal number of communities in the network (58). Low modularity suggests relative isolation of certain nodes. Thus, CBT-associated decrease in modularity may reflect a declining influence of certain “pernicious” nodes (e.g., symptom-driving hyperactive frontostriatal loops) on network dynamics, which are associated with improvements in OCD symptoms. Alternatively, the observed reduction in modularity in response to CBT associated with reduction in OCD symptoms may result from remodeling of brain network modular structure (i.e., shifting membership of nodes in the various modules). Another possible explanation is that reductions in modularity values reflect the development of cognitive compensatory mechanisms allowing for greater control over obsessions and compulsions and hence improved symptoms; indirect evidence for this comes from a study that found that, within individuals, reductions in functional modularity were associated with enhanced working memory (59).

### Clinical implications

In our sample, small-world network efficiency was a biomarker for longitudinal outcome in OCD subsequent to CBT. The effectiveness of CBT is well documented and beyond doubt, as exemplified in the current study that produced a high proportion of responders and remitters. Hence, a more useful clinical question for the application of biomarkers is not who will respond to treatment, but rather who will remain well and/or continue to improve after treatment. rsfMRI is a relatively easy to perform procedure that could be administered and replicated across sites, lending this method both to larger trials to confirm its utility in providing network connectivity measures to assist with clinical decision-making, and, if confirmed, ultimately in actual use in clinical settings. Contrary to this, predictors that involve neuropsychological factors may be less reliable than other, objective measurements such as resting-state brain connectivity due to the fact that they are highly effort-dependent and in some cases evaluator-dependent. These findings may also set the stage for testing different treatment durations to reduce risk of relapse. Adequate studies have not been conducted to determine the duration of treatment optimal for preventing relapse (60).

### Limitations

One limitation of this study is the small sample size, which may have constrained our ability to detect smaller changes in network measures and/or relationships with follow-up clinical status. This may have also accounted for the lack of significant node-level differences in graph-theory metrics pre- to post-treatment, for which we likely had insufficient power given the need to correct for multiple comparisons across 160 nodes. Another limitation, inherent to studies of a specific treatment modality that may restrict generalizability to the OCD population on the whole; it is possible that the OCD participants in this study may have “self-selected” for willingness to engage in difficult and intense exposure and response, which in turn likely results in significant reduction in symptoms.

## Conclusion

Brain organization characterized by less dense local functional connections (lower clustering coefficient and therefore lower small-worldness) prior to CBT confers greater maintenance of treatment gains. CBT increases network efficiency as it alleviates symptoms in most patients, but patients entering CBT with already high-network efficiency are at greater risk of relapse. Results show functional network efficiency as a biomarker of CBT response and relapse in OCD, which has potential implications for clinical decision-making and treatment selection. These results warrant further investigation and replication in a larger dataset.

## Conflict of Interest Statement

The authors declare that the research was conducted in the absence of any commercial or financial relationships that could be construed as a potential conflict of interest.

## Supplementary Material

The Supplementary Material for this article can be found online at http://journal.frontiersin.org/article/10.3389/fpsyt.2015.00074/abstract

## Footnotes

^1^http://www.fmrib.ox.ac.uk/fsl

^2^http://www.r-project.org/
