# Supplementary material for: Brain Connectivity and Prediction of Relapse after Cognitive-Behavioral Therapy in Obsessive–Compulsive Disorder
Source: Front Psychiatry. 2015 May 20;6:74. doi: 10.3389/fpsyt.2015.00074 (PMC4438601; doi:10.3389/fpsyt.2015.00074)
Supplement: Supplementary file 1 [file Data_Sheet_1.DOCX]

**Supplemental Information**

**Methods:**

*Cognitive-behavioral therapy (CBT) treatment for obsessive-compulsive disorder (OCD)*

The therapists followed the treatment protocol for Exposure and Response Prevention (ERP, a type of CBT for OCD) based on the manual by Kozak & Foa (1997) ([Kozak and Foa, 1997a](#_ENREF_2); [b](#_ENREF_3)). Each OCD participant was treated one-on-one by one of the two study therapists, who were experienced in ERP for OCD. The ERP sessions were 90 minutes each in duration, and were 5 days per week (Monday through Friday) for 4 weeks, for a total of 20 sessions. Participants were told that they could not be more than 10 minutes late to sessions or miss sessions. The study psychiatrist (JDF, or JL as backup) also met with each participant once weekly for 20 minutes. At each of these visits, if the participant was taking a stable dose of a serotonin reuptake inhibitor prior to enrollment, the psychiatrist assessed if he/she maintained the same dose of medication, and assessed for medication adherence. During the last week of treatment, the study therapist and study psychiatrist assisted the participant in referrals to outpatient treatment.

Outlines of the content of the therapy by session number are as follows:

*Sessions 1 and/or 2:*

- Rapport building
- Patient history
- Ascertainment of level of knowledge about OCD
- Facilitated discussion about the impact of OCD on their lives
- Description of OCD as a neurobehavioral disorder
- Demonstration of use of monitoring forms of symptoms, daily schedule, and structure
- Homework assignment: monitoring, or listing of obsessions and compulsions, and/or reading
- Reading material provided

*Sessions 2 and/or 3:*

- Homework review
- Identifying obsessions and compulsions
- Distinguishing obsessions and compulsions from other problems
- Description of the cycle of obsessions and compulsions
- Rationale and description of ERP
- Visual presentation and explanation of the course of OCD with and without response prevention
- Explanation of subjective units of distress (SUDS) graph
- Creation of hierarchy
- Homework assignment: self-monitoring of obsessions and compulsions, reading

*Sessions 4/5 through 18/19:*

- Review of significant events since last session
- Homework review
- Cognitive restructuring: a) to manage anxiety and resist compulsions if necessary; b) for reappraisal after exposure to consolidate learning; c) discussion of when it is *not* appropriate to use cognitive restructuring techniques (during exposures)
- Exposures exercises in session (in vivo or imaginal)
- Homework assignment: specific exposure exercises, self monitoring using SUDS

*Sessions 19/20:*

- Homework review
- Assessment of progress: review progress on hierarchy, discuss improvements overall with obsession thoughts and compulsive behaviors, improvements in overall functioning, improvements in overall anxiety and mood
- Current symptomatology and course of treatment: discuss and reinforce participant’s positive changes made during treatment (symptom reduction and better functioning)
- Inquiry of what participant learned from treatment
- Discuss relapse prevention: future plans for continuing treatment with outpatient CBT therapist; recognizing and dealing with symptoms as they arise; soliciting help from family or friends when necessary; increasing activities (e.g. work, school, relationships, hobbies); goals for 1 months, 3 months, 6 months, and 12 months in the future.
- Address termination
- Discussion of importance of follow-up treatment

**Results:**

**Table S1: Comorbid disorders**

| Comorbidities | | N |
| --- | --- | --- |
|  | None | 2 |
|  | MDD | 1 |
|  | SAD | 5 |
|  | GAD | 1 |
|  | Dysthymic disorder | 1 |
|  | Specific phobia | 1 |
|  | MDD and dysthymic disorder | 1 |
|  | GAD and PTSD | 1 |
|  | MDD and BDD | 1 |
|  | GAD and specific phobia | 1 |
|  | GAD and SAD and specific phobia | 1 |
|  | MDD and SAD and GAD | 1 |

*Post hoc analyses of pre- to post-treatment changes in graph theory metrics for unmedicated participants only*

After removing data from the three participants’ who were taking psychiatric medications during the intensive CBT phase, we obtained similar results as for the whole sample. Small-worldness AUC significantly increased from pre- to post-treatment (*P*<.0001). (Interestingly, 2 of the 3 medicated participants were the ones who had minimal increase, or else decrease, in small-worldness – see Fig. 4a, participants 13 and 15, respectively.) Mean clustering coefficient AUC also significantly increased from pre- to post-treatment (*P*<.0050). Modularity AUC significantly decreased from pre- to post-treatment (*P*=.012). There were no significant changes in local efficiency (*P*=.12), or global efficiency (*P*=.65).

*Full correlation analyses: Pre- to post-treatment changes in graph theory metrics*

In order to compare results to a previous pre- to post-treatment study in OCD of the effects of medication treatment on functional connectivity using graph theory ([Shin et al., 2014](#_ENREF_4)), we repeated our analyses using similar methodology of *full* correlation matrices rather than *partial* correlation matrices. Similar to the results for the partial correlation analysis, and results in the previous study, small worldness AUC increased from pre- to post-treatment, although only at trend level significance (*P*=0.063) (Fig. S2). Changes in small worldness were relatively consistent across the sample, with 11 of 17 participants demonstrating increases pre- to post-treatment, while 5 showed decreases pre- to post-treatment, and 3 were minimally changed. Mean clustering coefficient AUC increased from pre- to post-treatment, but results were not statistically significant (*P*=.12). There were no significant changes in AUC values for modularity (*P*=.35), global efficiency (*P*=.22), or local efficiency (*P*=.36). For the unmedicated sample, small worldness AUC increased from pre- to post-treatment, although only at trend level significance (*P*=0.067), as did mean clustering coefficient (*P*=0.067). There were no significant changes in AUC values for modularity (*P*=.45), global efficiency (*P*=.23), or local efficiency (*P*=.41).

**Supplementary Figures:**


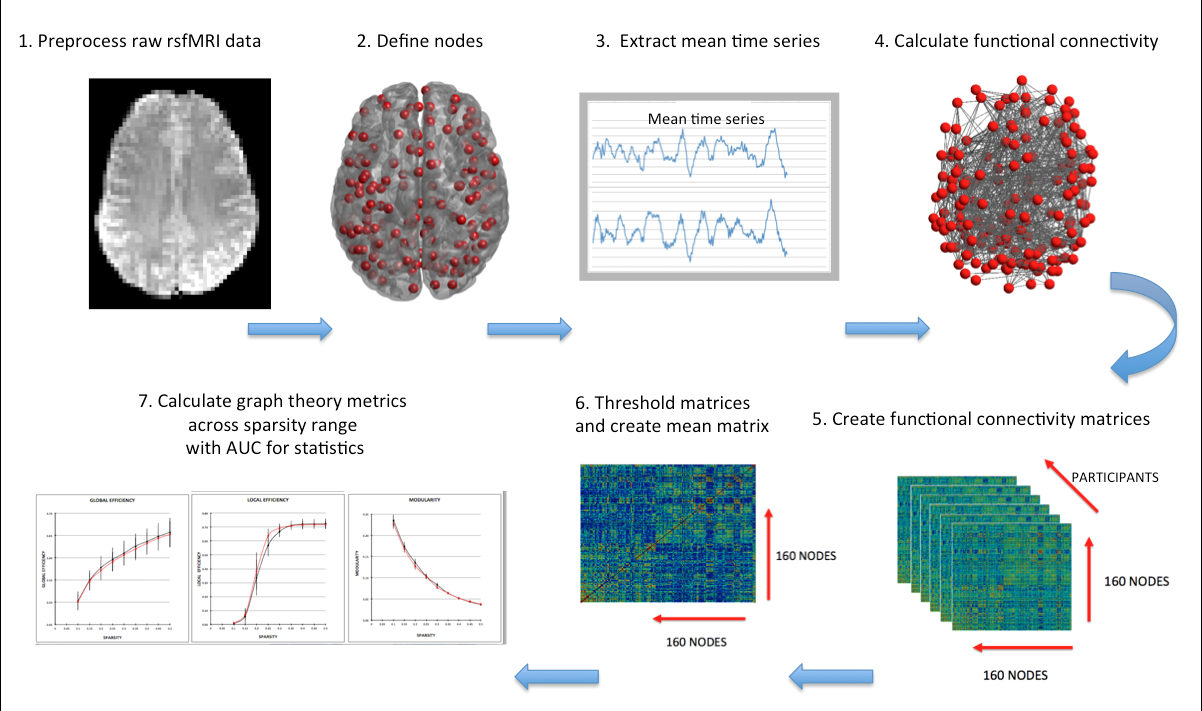


**Figure S1.** Flow chart of pre-processing and analysis steps. 1. Pre-processing of raw BOLD data. 2. Nodes were defined using centers from functional studies, as previously described ([Dosenbach et al., 2010](#_ENREF_1)). 3. Time series were extracted for each participant and averaged for each subject for each node. 4. Functional connectivity was calculated using the Brain Connectivity toolbox http://www.brain-connectivity-toolbox.net. 5. Partial and full functional connectivity matrices were calculated for each participant. 6. Mean weighted matrices for the pre-treatment and post-treatment conditions were calculated. 7. Graph theory metrics were calculated across a range of sparsities from 0.1 to 0.5 in steps of 0.05 as shown, then statistical comparisons between pre- and post-treatment were conducted using the area-under-the curve values for each participant for each metric. (Adapted from Shin et al., 2014 ([Shin et al., 2014](#_ENREF_4)).)


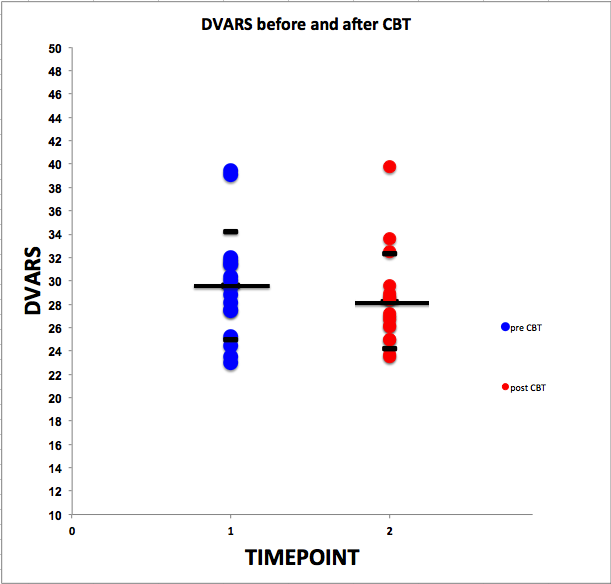


**Figure S2.**  Plot of motion estimate DVARS (root mean squared change in BOLD signal from volume to volume) for each participant pre- and post-CBT. Error bars are ± standard deviation. There were no significant differences in mean motion between the two time points (*P*=.33).


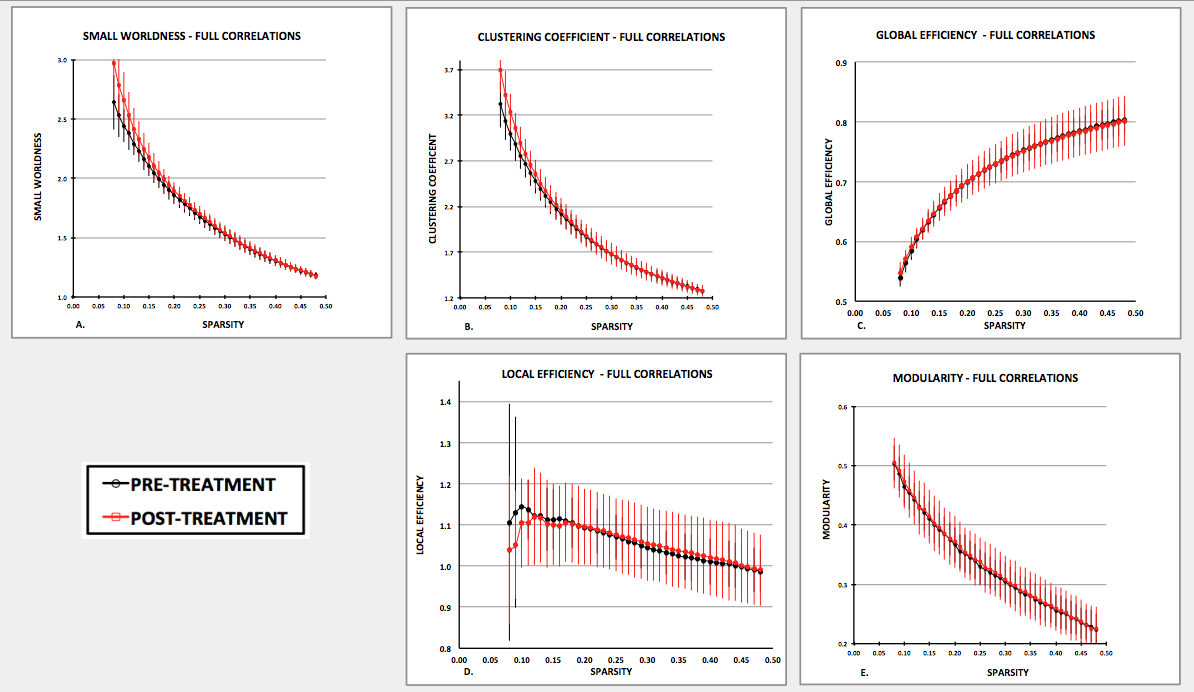


**Figure S3.**  Plot of graph theory metrics for full correlation matrices, across sparsity levels from 0.08 to 0.48: A. small worldness, B. clustering coefficient, C. global efficiency, D. local efficiency, E. modularity. Error bars are ± standard deviation


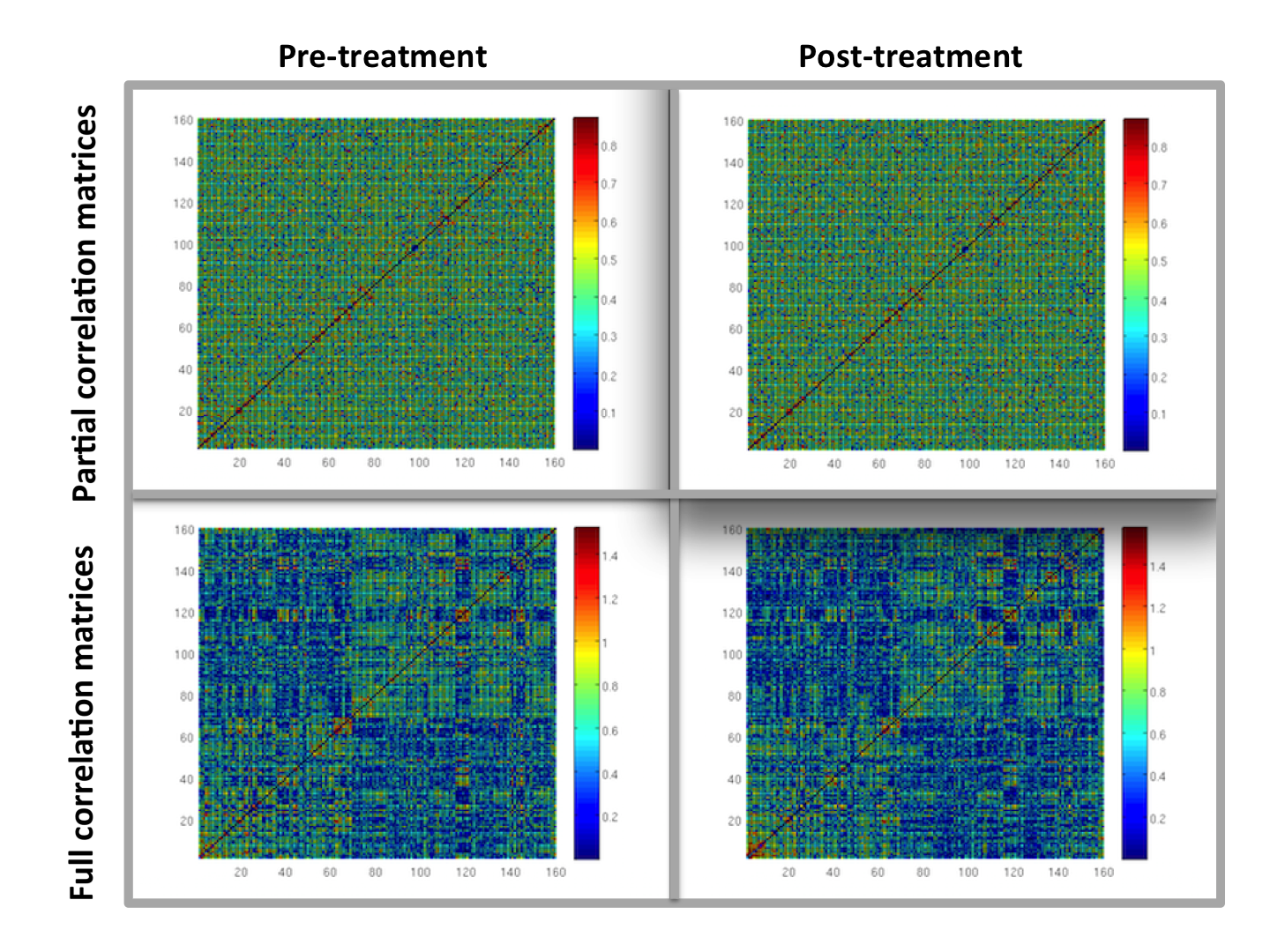


**Figure S4**. The mean correlation matrices pre- and post-treatment. The mean correlation matrix was calculated by averaging correlation matrices across participants. Partial correlation matrices are shown on top and full correlation matrices are shown on bottom.

**Table S2: Node-level results for pre- and post-CBT graph theory metrics**

| **Node** | **MNI coordinates** | **Metric** | **Direction^a^** | **Uncorrected *P* value^b^** |
| --- | --- | --- | --- | --- |
| R precentral gyrus | 60, 8, 34 | betweenness centrality | post>pre | .002 |
| R insular cortex | 33, -12, 16 | clustering coefficient | post>pre | .005 |
| L brainstem | -4, -31, -4 | local efficiency | post>pre | .005 |
| R angular gyrus | 44, -52, 47 | degree | pre>post | .006 |
| L precentral gyrus | -38, -15, 59 | clustering coefficient | post>pre | .006 |
| R precentral gyrus | 18, -27, 62 | local efficiency | post>pre | .006 |
| R occipital fusiform | 20, -78, -2 | degree | post>pre | .007 |
| L inf. frontal gyrus | -46, 10, 14 | betweenness centrality | post>pre | .007 |
| paracingulate gyrus | 0, 15, 45 | degree | pre>post | .010 |
| R middle frontal gyrus | 46, 28, 31 | betweenness centrality | pre>post | .010 |

Nodes results are shown for findings with uncorrected *P* values ≤.01

CBT = cognitive-behavioral therapy; MNI = Montreal Neurological Institute coordinates of center of 10 mm spherical nodes

^a^Direction of significant results: “post” = after CBT; “pre” = before CBT

^b^None of the nodal results passed False Discovery Rate (FDR) correction for multiple comparisons

**Supplementary References:**

Dosenbach, N.U., Nardos, B., Cohen, A.L., Fair, D.A., Power, J.D., Church, J.A., Nelson, S.M., Wig, G.S., Vogel, A.C., Lessov-Schlaggar, C.N., Barnes, K.A., Dubis, J.W., Feczko, E., Coalson, R.S., Pruett, J.R., Jr., Barch, D.M., Petersen, S.E., and Schlaggar, B.L. (2010). Prediction of individual brain maturity using fMRI. *Science* 329**,** 1358-1361. doi: 10.1126/science.1194144.

Kozak, M.J., and Foa, E.B. (1997a). *Mastery of obsessive-compulsive disorder: a cognitive-behavioral approach client workbook.* New York: Oxford University Press.

Kozak, M.J., and Foa, E.B. (1997b). *Mastery of obsessive-compulsive disorder: a cognitive-behavioral approach therapist guide.* New York: Oxford University Press.

Shin, D.J., Jung, W.H., He, Y., Wang, J., Shim, G., Byun, M.S., Jang, J.H., Kim, S.N., Lee, T.Y., Park, H.Y., and Kwon, J.S. (2014). The effects of pharmacological treatment on functional brain connectome in obsessive-compulsive disorder. *Biological Psychiatry* 75**,** 606-614. doi: 10.1016/j.biopsych.2013.09.002.
